# *Listeria monocytogenes* infection in multiple myeloma: A case report about a rare but lethal complication requiring heightened clinical vigilance

**DOI:** 10.1097/MD.0000000000045685

**Published:** 2025-11-07

**Authors:** Qiuyue Wu, Wenhui Liu, Tao Wu, Yang Liu

**Affiliations:** a Department of Hematology, The 940th Hospital of Joint Logistics Support Force of Chinese People’s Liberation Army, Lanzhou, Gansu, China.

**Keywords:** bortezomib, dexamethasone, lenalidomide, *Listeria monocytogenes*, multiple myeloma

## Abstract

**Rationale:**

Multiple myeloma (MM), ranking as the second most prevalent hematologic malignancy. Despite prolonged survival in MM due to novel therapies, infections remain a leading cause of mortality. While bacterial pneumonias and viral reactivations are well-documented, *Listeria monocytogenes (L monocytogenes*) (a rare but aggressive pathogen) is underrecognized in MM.

**Patient concerns:**

In this report, we report a 60-year-old male patient with newly diagnosed IgA-λ MM (Durie–Salmon stage IIIA, ISS stage III with 1q21 amplification) who developed high-grade fever (39.8 °C) and expressive aphasia during the second cycle of bortezomib–lenalidomide–dexamethasone induction therapy. Initial workup revealed neutrophilia (absolute neutrophil count 1.94 × 10⁹/L) with elevated inflammatory markers (C-reactive protein 118.14 mg/L). Cerebrospinal fluid analysis showed albuminocytologic dissociation (protein 1116.3 mg/L, glucose 1.77 mmol/L, chloride 114.75 mmol/L) without positive cultures. Contrast-enhanced brain MRI demonstrated neither abscess formation nor acute ischemia.

**Diagnoses:**

Repeated blood cultures drawn at fever onset, *L monocytogenes* bacteremia was confirmed 48 hours after-afebrile through blood culture. The patient has been diagnosed MM with bacteremia and meningitis caused by *L monocytogenes* infection.

**Interventions:**

Empiric meropenem was initiated promptly, achieving defervescence within 72 hours. Upon availability of antimicrobial susceptibility testing (intravenous penicillin G 8 million units every 8 hours), with documented blood culture clearance at 72 hours.

**Outcomes:**

On the second day of medication, the patient’s aphasia symptoms were resolved, the fever subsided on the 3rd day, and the blood culture turned negative on the 10th day. The patient completed a 21-day targeted antibiotic course without neurologic sequelae and was discharged with planned resumption of anti-myeloma therapy.

**Lessons:**

This case underscores the need for early empiric coverage for *Listeria* in MM patients with unexplained fever or neurological symptoms, particularly given its high mortality in immunocompromised hosts.

## 1. Introduction

Multiple myeloma (MM), ranking as the second most prevalent hematologic malignancy, has transitioned to a chronic disease model owing to therapeutic advancements that have significantly extended patient survival. Nevertheless, pathological immunoglobulin overproduction by malignant plasma cells induces severe clinical manifestations, including osteolytic lesions, renal impairment, and heightened infection susceptibility. Notably, infectious complications represent the primary contributor to both morbidity and mortality in MM, frequently precipitating acute clinical deterioration in otherwise stable patients.

The spectrum of MM-associated infections predominantly comprises bacterial pathogens (typically causing pneumonia and bacteremia) and viral agents (notably influenza and herpes zoster). Of particular concern are infections with *Listeria monocytogenes (L monocytogenes*), an atypical but increasingly recognized pathogen in this immunocompromised population. Despite its relative rarity, *L monocytogenes* infection carries substantial mortality risk in MM patients, emphasizing the critical need for prompt diagnosis and targeted antimicrobial therapy. This report details a representative case of listeriosis in an MM patient, aiming to heighten clinical vigilance regarding this potentially catastrophic complication.

## 2. Clinical data overview

### 2.1. Case presentation

The patient is an elderly married male from a rural area with a previously stable social life and good psychological status, supported by a well-established family support system. His usual nutritional status is acceptable, and he has no history of smoking or alcohol consumption. Vaccinations were administered according to the local schedule without significant omissions. There is no clear history of residence in endemic areas or exposure to contaminated water, no past recurrent or chronic infections, and no recent trauma, surgery, or invasive procedures. He was initially admitted to a local hospital on May 3, 2024, presenting with intermittent sacrococcygeal pain and dysuria without apparent cause. His symptoms included fever, productive cough, abdominal pain and distension, diarrhea, chest tightness, shortness of breath, and dyspnea. While he denied episodes of nosebleeds or gingival bleeding, and he didn’t exhibit systemic skin and mucous membrane bleeding manifestations.

### 2.2. Diagnostic evaluation and initial findings

Blood tests revealed: white blood cell count 3.76 × 10^9^/L, hemoglobin 87 g/L, and platelet count 125 × 10^9^/L. Biochemical analysis showed elevated globulin at 55.7 g/L. Lumbar MRI demonstrated degenerative changes in the lumbar spine and facet joints, mild anterior spondylolisthesis of L5 with old compression fractures, disc bulging at L2/3-L5/S1 levels, and interspinous ligamentitis. Bone marrow aspiration examination indicated active karyocyte proliferation with plasma cells accounting for 57% (including 23.5% juvenile forms). Morphological assessment revealed pleomorphic plasma cells with abundant dark blue foamy cytoplasm, eccentrically located round nuclei with coarse chromatin pattern, and frequent bi-/multinucleated forms, consistent with MM. Serum protein electrophoresis detected an M-protein spike of 36.32 g/L (β1-globulin region, 49.1%), with elevated IgA (35.5 g/L) and lambda light chains (10.55 g/L). Immunofixation electrophoresis confirmed IgA-λ type monoclonal protein. Serum and urine free light chain levels were within normal ranges.

### 2.3. Further specialized testing

The patient was transferred to our hospital on May 16, 2024 for further management. Repeat bone marrow examination showed active hyperplasia with 53.6% abnormal plasma cells (49.6% primitive/naive forms). Immunophenotyping demonstrated an abnormal monoclonal plasma cell population (7.52% of nucleated cells) expressing CD138, CD38, CD56, and cLambda, while negative for CD19 and cKappa. Serum protein electrophoresis confirmed M-protein (36.32 g/L) of IgA-λ type. Cytogenetic analysis showed normal karyotype (46, XY). FISH testing identified 1q21 amplification (17% of cells with > 2 copies, 83% with 2 copies). Based on these findings, the patient was diagnosed with IgA-λ type MM, Durie-Salmon stage IIIA, ISS stage III, with 1q21 amplification indicating high-risk disease.

### 2.4. Treatment course

**Figure 1.**
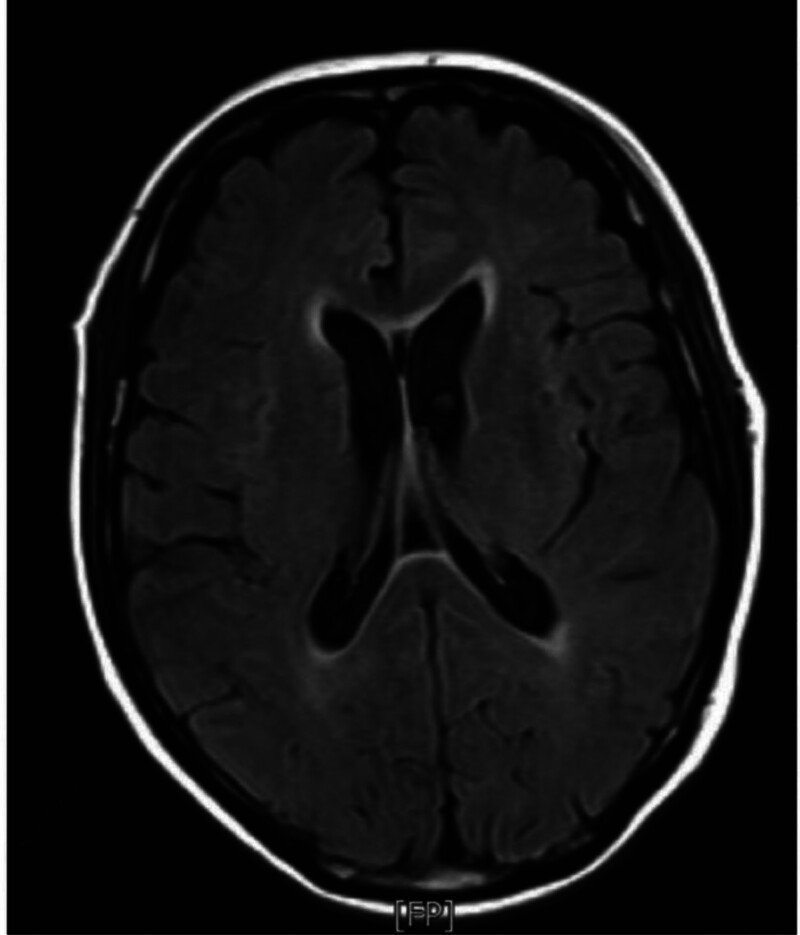
Magnetic resonance imaging (MRI) T1 weighted sequences of the brain (June 6, 2024). MRI reveals abnormal hyperintense in diploe of frontal, parietal, temporal bone, and occipital bones, leukodystrophy signal in both sides of the lateral ventricles.

**Figure 2.**
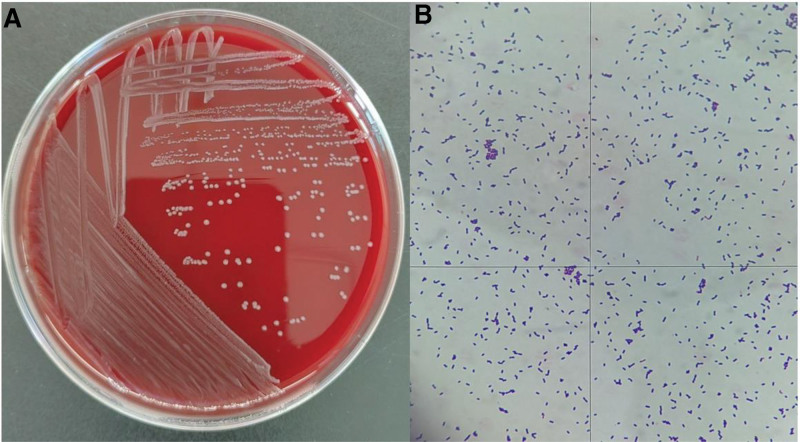
Blood culture (A) and gram stain 1000× (B). The blood plate revealed that the bacterial colonies were small, round, smooth, gray, and had a narrow β lytic ring (A), which revealed gram-positive microbacilli, bacterial cells are unbranched, spore-free, 0.5 to 2 μm long, 0.4 to 0.5 μm wide, and single and short chains (B).

The patient began combination chemotherapy with bortezomib (2 mg), lenalidomide (25 mg), and dexamethasone (8 mg) (BRD regimen). On June 5, 2024 (day 15 of cycle 2), he developed acute fever (37.8 °C) and aphasia. During the acute phase of the illness, we immediately discontinued chemotherapy and initiated blood cultures and infection marker monitoring. To rule out neurological disorders, a head CT, MRI, and cerebrospinal fluid analysis (CSF) were performed. The cranial MRI findings only revealed primary changes related to MM, essentially ruling out neurological symptoms caused by organic brain lesions (Fig. 1). Additionally, we requested a neurology consultation. Based on the head CT, MRI, and CSF biochemical results, neurological disorders were ruled out. Following empirical treatment guidelines, we promptly initiated intravenous meropenem (1 g every 8 hours) for the patient. Over the subsequent 2 days, he experienced persistent fever (peak 39.8 °C) (Table 1). On day 9 of symptom onset, blood cultures returned positive for *L monocytogenes*, suggesting possible bacteremia and meningitis (Fig. 2). Antibiotic therapy was switched to intravenous penicillin G (8 million units every 8 hours). Repeat blood cultures were collected alongside CSF analysis and culture. CSF culture showed no growth, and subsequent blood cultures cleared. CSF biochemical analysis revealed elevated protein with decreased glucose and chloride levels (Table 2). The patient’s condition stabilized, and he was discharged with plans to complete a 3-week course of intravenous penicillin (through July 3). Following this infection, the patient’s condition was reassessed, and the dexamethasone dosage was reduced from the previous 8 mg to 4 mg. During long-term follow-up, the patient’s condition remained stable with continuous BRD therapy. A reexamination on February 17, 2025, confirmed complete remission. The treatment has now been switched to lenalidomide for maintenance. Additionally, during this period, the patient did not experience any recurrent infections, and no significant abnormalities were detected in laboratory tests such as complete blood count.

**Table 1:** Blood examination results from hospital admission to discharge.

Data result	May 19	June 4	June 5	June 6	June 8	June 10	June 12	June 15
WBC (10^9^/L)	3.95	3.02	2.59	2.07	2.85	3.99	2.29	6.41
NEU (10^9^/L)	1.96	1.82	1.94	1.77	2.06	2.87	1.34	5.32
LY (10^9^/L)	1.65	0.73	0.37	0.13	0.56	0.80	0.64	0.84
MONO (10^9^/L)	0.26	0.44	0.28	0.17	0.22	2.87	0.29	5.32
MONO (%)	6.6	14.4	10.6	8.2	7.8	7.5	12.6	3.5
IL-6 (pg/mL)	–	–	217.1	116.0	–	–	–	–
PCT (ng/mL)	–	–	3.190	4.100	–	–	–	–
CRP (mg/L)	<3.01	–	–	118.14	–	10.50	–	–
Temp (°C)	Highest 36.3	36.5	39.8	39.0	37.4	36.8	36.9	36.3
	Lowest 36.0	36.1	38.5	36.1	37.2	36.0	36.2	36.0
Blood culture	–	–	(−)	(−)	(−)	(−)	*L monocytogenes*	(−)

CRP = C-reactive protein, IL-6 = interleukin-6, *L monocytogenes = Listeria monocytogenes*, LY% = lymphocyte percentage, MONO% = monocyte percentage, MONO = monocyte, NEU% = neutrophil percentage, PCT = procalcitonin, temp = temperature, WBC = white blood cell.

**Table 2:** Cerebrospinal fluid examination result.

CSF	Result
Bacteria	Negative
Color	Pale yellow
Character	Limpid
WBC (10^6^/L)	91
NEU (%)	19.2
LY (%)	80.8
Protein (mg/L)	1116.3
Chlorine (mmol/L)	114.75
Glucose (mmol/L)	1.77

CSF = cerebrospinal fluid, LY = lymphocyte, NEU% = neutrophil percentage, WBC = white blood cell.

## 3. Discussion

### 3.1. The characterize of *L monocytogenes*

*L monocytogenes*, a gram-positive bacterium, is a foodborne pathogen that mediates listeriosis as an opportunistic pathogen. *L monocytogenes* is the only *Listeria* species that has been recognized as a human pathogen.^[1]^ The incidence of listeriosis in the average annual population is 0.29 cases per 100,000, of which 74% of nonpregnant patients aged < 65 years are in an immunocompromising condition, most commonly immunosuppressive therapy or malignancy. However, it carries substantial mortality at approximately 20% to 30%.^[2]^ This infection especially occurs in immunocompromised and elderly individuals, and in maternal–fetal infections. After eating food contaminated by the host, *L monocytogenes* encounters the intestinal epithelium, traverses the intestinal epithelium, and enters the lymph nodes and bloodstream, causing bacteremia. It can also cross the blood–brain barrier, causing nervous system diseases, such as meningoencephalitis. The patient presented with headache, fever, disturbance of consciousness, and focal neurological signs, including cranial nerve palsy.^[3]^ Infection often occurs in the brain tissue and can be found intra- and extracellularly in the brain parenchyma, blood vessels, and meninges.^[4]^ Most *Listeria* were observed in the brain parenchyma rather than in the CSF. For auxiliary examination, blood cultures were usually positive and CSF cultures were mainly negative. In the CSF, there were approximately 100 cells/mm^3^, mostly neutrophils, as well as elevated protein concentration and normal glucose levels, when CSF culture was positive, it was probably due to rupture of the abscess. Our findings corroborated this pattern. Thus, the diagnosis of *L monocytogenes* central invasion in patients with negative CSF cultures but symptoms remains a challenge, but treatment should also be initiated based on the specificity of patients with MM.

### 3.2. Therapeutic considerations

For the treatment of *L monocytogenes* infection, urgent administration of adequate antimicrobial treatment is key to preventing complications, death, and long-lasting sequelae in human listeriosis. Beta-lactams, gentamicin, and co-trimoxazole are advised over other antibiotics for bacteremia and neurolisteriosis during infections.^[5]^ β-Lactam antibiotics, or in combination with an aminoglycoside, are considered the gold standard for the treatment of *L monocytogenes* infections. Ampicillin or penicillin G combined with an aminoglycoside, classically gentamicin, is the most commonly prescribed treatment for listeriosis, and a trimethoprim/sulfamethoxazole combination can be used as an alternative treatment.^[6]^ It is essential to coverage the treatment of central nervous system symptomatic MM with negative CSF culture. According to the Infectious Diseases Society of America and European Society of Clinical Microbiology and Infectious Diseases bacterial meningitis treatment guidelines, adult neurolisteriosis should be treated with 12 µg of β-lactam antibiotics per day for at least 21 days.^[7]^ Our case is a demonstration that *L monocytogenes* is also susceptible to meropenem.

### 3.3. MM-specific susceptibility risks

#### 3.3.1. Disease-related factors

MM is a heterogeneous malignancy characterized by infection and is a common complication. Owing to the cumulative effects of disease, treatment, and host-related factors, infection remains the leading cause of morbidity and mortality in patients with MM.^[8]^ In particular, newly diagnosed MM patients have an increased susceptibility to infection because of the cumulative effects of various factors.^[9]^

#### 3.3.2. Treatment-related factors

In addition to the disease itself, based on the function of monocytes and macrophages defending against *L monocytogenes*, some targeted drugs for treating MM are directly related to infection.

CD38 is an important target for the treatment of MM, which is highly expressed by myeloma cells, it is also expressed by activated macrophages and appears to play an important role in *Listeria* defense. Khan et al found that patients treated with daratumumab, a common human immunoglobulin G1κ monoclonal antibody directed against CD38, experienced a 75-fold listeriosis risk compared with all other patients with MM.^[10]^ Toshiyuki et al^[11]^ also reported a case of a patient with MM using another monoclonal antibody directed against CD38, isatuximab, which caused *Listeria* infection. *Listeria* has been demonstrated to be related to immunosuppressive therapy, and the use of BRD chemotherapy drugs may increase susceptibility to *Listeria* infection. Bici et al^[12]^ found that the rate of infection with BRD-based chemotherapy for newly diagnosed MM, including pneumonia and herpes, was low, with only 14.5% in their retrospective single-center cohort study. There are few reports and studies on *Listeria* infection in patients with MM receiving BRD-based chemotherapy. For BRD chemotherapeutic treatment, only dexamethasone has been shown to be associated with *Listeria* infections. Compared to other bacterial meningitis cases, Brouwer et al^[13]^ found that dexamethasone is detrimental to *L meningoencephalitis*. Glucocorticoids were discontinued over time. Bortezomib is a proteasome inhibitor which causes reactivation of the virus increasing the risk of virus infection.^[2,14]^ Among novel anti-MM drugs for treating relapsed/refractory MM, the risk of high-grade infection posed by lenalidomide may be the highest.^[15]^ Our patient only used the BRD chemotherapy scheme after newly diagnosed MM, so we suspect that, except for the disease itself, the adverse reactions to the drugs of the BRD chemotherapy scheme are the main reason for the infection.

## 4. Conclusion

Our study highlights the elevated risk of *L monocytogenes* infection in newly diagnosed MM patients undergoing BRD chemotherapy, particularly associated with dexamethasone-induced immunosuppression. Our findings demonstrate that neuroinvasive listeriosis may present with sterile CSF cultures despite characteristic biochemical alterations (elevated protein > 1g/L) and immediate meropenem initiation followed by penicillin de-escalation represents an effective therapeutic strategy.

As *L monocytogenes* is an opportunistic foodborne pathogen, patients with MM in hospitals should avoid unhygienic food and strictly avoid high-risk foods (unpasteurized dairy, processed meats) during neutropenic phases. Once patients gain the infection, their condition will be dangerous; therefore, supervising physicians need to identify early and pay high attention. When listeria infection is suspected, it is critical to mandatory blood cultures and CSF polymerase chain reaction when central nervous system symptoms occur, regardless of CSF culture status. At the same time, prompt glucocorticoid discontinuation while maintaining proteasome inhibitor/IMiDs if clinically feasible. Meropenem can combat Listeria while covering flora with similar symptoms when the type of infection is uncertain. However, there are still some clinical challenges. How to diagnose those patients with *L monocytogenes* central invasion with negative CSF cultures. Optimal antimicrobial selection and duration in hematological patients is a challenge and there are no specific guidelines. Pathogenetic contributions of specific BRD components, risk-benefit analysis of chemotherapy interruption versus continuation during active infection.

## Author contributions

**Data curation:** Wenhui Liu.

**Methodology:** Yang Liu.

**Writing – original draft:** Qiuyue Wu.

**Writing – review & editing:** Tao Wu.
